# Cardiac rehabilitation, physical activity, and the effectiveness of activity monitoring devices on cardiovascular patients: an umbrella review of systematic reviews

**DOI:** 10.1093/ehjqcco/qcad005

**Published:** 2023-01-23

**Authors:** Hila Ariela Dafny, Stephanie Champion, Lemlem G Gebremichael, Vincent Pearson, Jeroen M Hendriks, Robyn A Clark, Maria Alejandra Pinero de Plaza, Aarti Gulyani, Sonia Hines, Alline Beleigoli

**Affiliations:** Caring Futures Institute (CFI), College of Nursing and Health Sciences, Flinders University, Sturt Road, Bedford Park, Adelaide, SA 5042, Australia; Mparntwe Centre for Evidence in Health, Flinders University: A JBI Centre of Excellence, Alice Springs, Northern Territory 0871, Australia; Caring Futures Institute (CFI), College of Nursing and Health Sciences, Flinders University, Sturt Road, Bedford Park, Adelaide, SA 5042, Australia; Mparntwe Centre for Evidence in Health, Flinders University: A JBI Centre of Excellence, Alice Springs, Northern Territory 0871, Australia; Caring Futures Institute (CFI), College of Nursing and Health Sciences, Flinders University, Sturt Road, Bedford Park, Adelaide, SA 5042, Australia; Mparntwe Centre for Evidence in Health, Flinders University: A JBI Centre of Excellence, Alice Springs, Northern Territory 0871, Australia; Caring Futures Institute (CFI), College of Nursing and Health Sciences, Flinders University, Sturt Road, Bedford Park, Adelaide, SA 5042, Australia; Caring Futures Institute (CFI), College of Nursing and Health Sciences, Flinders University, Sturt Road, Bedford Park, Adelaide, SA 5042, Australia; Mparntwe Centre for Evidence in Health, Flinders University: A JBI Centre of Excellence, Alice Springs, Northern Territory 0871, Australia; Centre for Heart Rhythm Disorders, University of Adelaide and the Royal Adelaide Hospital, Adelaide, SA, Australia; Caring Futures Institute (CFI), College of Nursing and Health Sciences, Flinders University, Sturt Road, Bedford Park, Adelaide, SA 5042, Australia; Mparntwe Centre for Evidence in Health, Flinders University: A JBI Centre of Excellence, Alice Springs, Northern Territory 0871, Australia; Caring Futures Institute (CFI), College of Nursing and Health Sciences, Flinders University, Sturt Road, Bedford Park, Adelaide, SA 5042, Australia; Mparntwe Centre for Evidence in Health, Flinders University: A JBI Centre of Excellence, Alice Springs, Northern Territory 0871, Australia; National Health and Medical Research Council, Transdisciplinary Centre of Research Excellence in Frailty and Healthy Ageing, Adelaide, SA, Australia; Caring Futures Institute (CFI), College of Nursing and Health Sciences, Flinders University, Sturt Road, Bedford Park, Adelaide, SA 5042, Australia; Mparntwe Centre for Evidence in Health, Flinders University: A JBI Centre of Excellence, Alice Springs, Northern Territory 0871, Australia; Flinders Rural and Remote Health, NT. College of Medicine and Public Health, Flinders University, Alice Springs, NT, Australia; Caring Futures Institute (CFI), College of Nursing and Health Sciences, Flinders University, Sturt Road, Bedford Park, Adelaide, SA 5042, Australia; Mparntwe Centre for Evidence in Health, Flinders University: A JBI Centre of Excellence, Alice Springs, Northern Territory 0871, Australia

**Keywords:** Activity-monitoring, Cardiac rehabilitation, Physical activity, Umbrella review, Randomized controlled trials

## Abstract

**Aims:**

To consolidate the evidence on the effectiveness of activity-monitoring devices and mobile applications on physical activity and health outcomes of patients with cardiovascular disease who attended cardiac rehabilitation (CR) programmes.

**Methods and results:**

An umbrella review of published randomized controlled trials, systematic reviews, and meta-analyses was conducted. Nine databases were searched from inception to 9 February 2022. Search and data extraction followed the JBI methodology for umbrella reviews and PRISMA guidelines. Nine systematic reviews met the inclusion criteria, comparing outcomes of participants in CR programmes utilizing devices/applications, to patients without access to CR with devices/applications. A wide range of physical, clinical, and behavioural outcomes were reported, with results from 18 712 participants. Meta-analyses reported improvements in physical activity, minutes/week [standardized mean difference (SMD) 0.23, 95% confidence interval (CI) 0.10–0.35] and activity levels (SMD 0.29, 95% CI 0.07–0.51), and a reduction in sedentariness [risk ratio (RR) 0.54, 95% CI 0.39–0.75] in CR participants, compared with usual care. Of clinical outcomes, the risk of re-hospitalization reduced significantly (RR 0.49, 95% CI 0.27–0.89), and there was reduction (non-significant) in mortality (RR 0.27, 95% CI 0.05–1.54). From the behavioural outcomes, reviews reported improvements in smoking behaviour (RR 0.87, 95% CI 0.67–1.13) and total diet quality intake (RR 0.79, 95% CI 0.66–0.94) among CR patients.

**Conclusions:**

The use of devices/applications was associated with increase in activity, healthy behaviours, and reductions in clinical indicators. Although most effect sizes indicate limited clinical benefits, the broad consistency of the narrative suggests devices/applications are effective at improving CR patients’ outcomes.

## Introduction

Cardiovascular disease (CVD) was responsible for 17.7 million of the 55 million deaths in 2017, globally.^1^ After a first acute CVD event, recurrence is common and is associated with disease progression and higher morbidity and mortality with an 18% absolute risk of event recurrence or death within 12 months.^2^ Thus, secondary prevention has paramount importance in reducing the burden of CVD.

Cardiac rehabilitation (CR) is a structured multidisciplinary secondary prevention programme that supports successful risk factor management and re-integrate patients with CVD into their former family, social, and work-related lives after an acute event or procedure.^3,4^ Clinical guidelines strongly recommend referring patients at high risk of an acute CVD-related event to CR.^3,5^ Exercise training is a critical component of CR and is often offered in supervised, clinic-based CR programmes either in isolation or combined with other risk factor modification core components.^3,4^ For people with coronary heart disease, there is moderate to high certainty evidence that exercise-based CR is associated with a reduction in 6–12-month all‐cause mortality [risk ratio (RR) 0.87, 95% confidence interval (CI) 0.73–1.04], myocardial infarction (RR 0.72, 95% CI 0.55–0.93), and all‐cause hospitalization (RR 0.58, 95% CI 0.43–0.77) and little to no difference in risk of cardiovascular mortality (RR 0.88, 95% CI 0.68–1.14).^6^ For patients with heart failure (HF) (either with reduced or preserved ejection fraction) there is low to moderate certainty evidence that CR reduces overall hospital admissions up to 1 year of follow‐up (RR 0.70, 95% CI 0.60–0.83) and HF‐specific hospitalization (RR 0.59, 95% CI 0.42–0.84) with no effect on mortality.^6^ Moreover, after CR, a clinically important improvement in health‐related quality of life may be evident. These effects are consistent across different models of exercise-based CR delivery (centre vs. home‐based) and across different exercise doses and modalities (aerobic training alone vs. aerobic plus resistance programmes).^7^

Despite all these benefits, CR is under-utilized with only 20–50% of eligible patients attending a CR programme globally.^6^ Reasons for this gap in CR evidence translation into practice include low medical referral, travel distance to the centres where CR programmes are traditionally delivered, inconvenient programme scheduling, lack of social support, and inappropriateness/lack of tailoring of CR delivery including the exercise component.^8,9^ The rapid adoption of telehealth-enabled delivery modes after suspension of in-person CR programmes during the COVID-19 pandemic demonstrated that telehealth offers a solution to some of these barriers.^10,11^ However, the delivery of exercise training and physical activity counselling was the most affected among the CR components. The low availability of telemonitoring technologies incorporated in the programmes was pointed out as one of the reasons for low exercise training participation during COVID.^10,11^ However, despite the existence of several systematic reviews on integrating technologies^12–16^ such as sensors, pedometers, accelerometers, wearable activity-monitoring devices, and smart phone applications to CR programmes, the effectiveness of these trackers is not well established. This umbrella review collates and consolidate the evidence from systematic reviews and meta-analyses of randomized controlled trials (RCTs) investigating the effectiveness of the use of activity-monitoring devices and mobile applications within CR programmes compared with non-use of devices and applications on (i) physical activity levels and (ii) clinical outcomes.

## Methods

**Figure 1:**
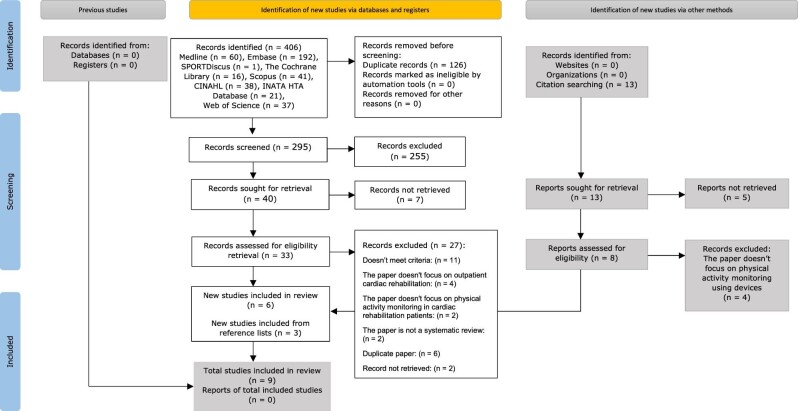
PRISMA flow diagram of search and study selection process.

An umbrella review was conducted following JBI review guidelines. The protocol of this umbrella review was registered on PROSPERO in 2021 CRD42022298877 and published in JBI Evidence Synthesis, where the details on the eligibility criteria, databases sources, search strategy, selection and data collection process, items and risk of bias assessments, effect measures, synthesis methods, reporting bias, and certainty assessments can be accessed.^17^ This review follows the guideline for the Preferred Reporting Items for Systematic Reviews and Meta-Analyses (PRISMA) (*Figure**1*).^18^ See *Figure* *1* for a PRISMA flow diagram of search and study selection process and Supplementary material online, *Table 3* for a list of excluded studies and reasons for exclusion at full-text review.

### Search

Articles published in English from database inception to the 9 February 2022 were searched. Following the initial search on Medline (Ovid) and the Cochrane Database of Systematic Reviews, all identified keywords and index terms were translated to the other searched databases. Searches were conducted in MEDLINE (Ovid), Embase (Ovid), Sport Discus, Cochrane Database of Systematic Reviews, Scopus (Elsevier), CINAHL (EBSCO), the International Network of Agencies for Health Technology Assessment (INAHTA), Epistemonikos, and the Web of Science. Citation details were imported into the JBI System for the Unified Management, Assessment and Review of Information.^19^ Primary outcomes of focus were physical activity outcomes assessed using activity-monitoring devices and mobile applications and reported as steps per day, heart rate, energy expenditure, sedentary time, minutes of activity per day, or intensity of the activity. Secondary outcomes included clinical health outcomes measured using activity-monitoring devices and mobile applications, such as mortality rate, the incidence of myocardial infarction, revascularization, and hospital admissions of >24 h.

### Quality assessment

The methodological quality of the included reviews was independently assessed by two reviewers (H.D., S.C., L.G., V.P., and A.B.), using the JBI SUMARI instrument for systematic reviews.^20^

### Data extraction and synthesis

Data was extracted by two reviewers using the standardized JBI data extraction tool.^20^ Characteristics of the included reviews (number of studies, number, sex, and age of participants, diagnosis, duration, follow-up, number of events, type of wearables, description of the intervention, and types of outcomes) were presented (Supplementary material online, *Table 2*). Where meta-analysis was performed, effect size (risk ratio, odds ratio, hazard ratio, or standardized weighted difference) for outcome measures for the intervention and control groups were extracted and presented in summary tables and graphs. This umbrella review collated five meta-analyses presented in the included articles. The effect size measures and confidence intervals, heterogeneity scores, chi-square statistics, degrees of freedom, and *Z*-scores between the factor and outcome were reported when they were provided.

## Results

### Study characteristics

Across the systematic review cohorts, the median age was 70 years of age (range: 42–82 years), and the majority of participants were male (70–78%). The total number of studies included in each review ranged from 8 to 40, with 545 to 6480 participants. Of the nine included studies (*Figure**1*: PRISMA flow diagram), four used mobile applications, four used activity-monitoring devices, and one used both mobile and activity-monitoring devices as an intervention for physical activity levels and health outcomes. Seven of the nine studies (78%) reported on physical activity and health outcomes, while two studies assessed physical activity and other behavioural outcomes only (Supplementary material online, *Table 2*).

### Assessment of quality

Overall, all the systematic reviews had a high level of methodological quality. *Table* *1* shows the outcomes of the methodological assessment using the JBI tool.^20^ Of the nine reviews included, five were rated high quality, two were rated moderate quality,^6,21^ and two were rated low quality.^15,22^

**Table 1:** The quality of evidence in systematic review based on JBI SUMARI tool^20^

Authors	Review question clear	Inclusion criteria appropriate	Search strategy appropriate	Search for studies adequate?	Criteria for appraisal appropriate?	Critical appraisal by two or more reviewers?	Methods to minimize errors in extraction	Methods to combine studies appropriate	Likelihood of publication bias assessed	Recommendations supported by data	Directives for new research appropriate?	Total score	%
Akinosun (2021)^23^	Yes	Yes	Yes	Yes	Yes	Yes	Yes	Yes	Yes	No	Yes	10/11	90
Hannan (2019)^13^	Yes	Yes	Yes	Yes	Yes	Yes	Yes	Yes	Unclear	Yes	Yes	10/11	90
Su (2020)^24^	Yes	Yes	Yes	Yes	Yes	Yes	Yes	Yes	Unclear	Yes	Yes	10/11	90
Indraratna (2020)^25^	Yes	Yes	Yes	Yes	Yes	Yes	Yes	Yes	No	Yes	Yes	10/11	90
van Veen (2017)^26^	Yes	Yes	Yes	Yes	Yes	Yes	Yes	Yes	No	Yes	Yes	10/11	90
Marin (2019)^21^	Yes	Yes	Yes	Yes	Yes	Yes	Yes	No	Yes	No	Yes	9/11	82
Dibben (2018)^6^	Yes	Yes	Yes	Yes	Yes	Unclear	Yes	Yes	Unclear	Yes	Yes	9/11	82
Rawstorn (2016)^15^	Yes	Yes	Unclear	Yes	Unclear	Unclear	Yes	Yes	Yes	Yes	Yes	8/11	73
Batalik (2020)^22^	Yes	Yes	Yes	No	Yes	Unclear	Unclear	Yes	No	Yes	Yes	7/11	64

Overall % score = sum of all %.

The criterion that was not available or not clearly reported by most (*n* = 6) of the included reviews was the assessment of the likelihood of publication bias (criterion 9). However, other sources of bias were considered in the reviews. For example, Hannan *et al.* (2019) described potential bias arising from study attrition, unrepresentative samples, and randomization bias.^13^

### Overlapping reviews

A degree of overlap across the included reviews was anticipated as the publications that met the inclusion criteria examined had similar methodologies, exposures, and outcomes. The percentage of overlap between reviews, defined as the percentage of primary publications that were included in more than one review as a proportion of the total number of included publications, was calculated to be 19%. As this value does not account for the publications that appear in more than two reviews, the corrected cover area (CCA) was calculated using the method proposed by Pieper *et al.* (2013).^27^ The CCA was calculated to be 4%. This is a slight overlap which is unlikely to have an impact on the conclusions of the umbrella review.^27^ In light of the overlap in primary reviews, where there are multiple measures for an outcome of interest, the findings from the largest systematic review will be stated.

### Meta-analyses of RCTs

#### The impact of devices/apps as part of CR on physical activity outcomes

Five of the nine reviews reported meta-analyses conducted on physical activity.^6,13,15,23,24^ There were considerable variations in the physical activity metrics, and the methods used to analyse and report the outcomes. Su *et al.* (2020), Hannan *et al.* (2019), and Dibben *et al.* (2018) reported meta-analysis on daily step counts, collected using pedometers and accelerometers.^6,13,24^ Su *et al.* (2020) and Dibben *et al.* (2018) reported minutes of activity, and Dibben *et al.* (2018) also reported on activity at different levels of intensity.^6,24^ Akinosun *et al.* (2021) reported on minutes of activity and minutes of inactivity, with data collected using mobile apps and sensors,^23^ and Rawstron *et al.* (2016) outlined outcomes of activity level, reported as daily step counts or weekly energy expenditure, monitored with telephone, smartphone, mobile applications, computer, internet, and biosensors.^15^ The inconsistency in modes of data collection limits the number of reviews that could be included in meta-analyses, and insufficient detail was provided in the reviews to calculate average step counts and time spent in activity across the included RCTs.

**Figure 2:**
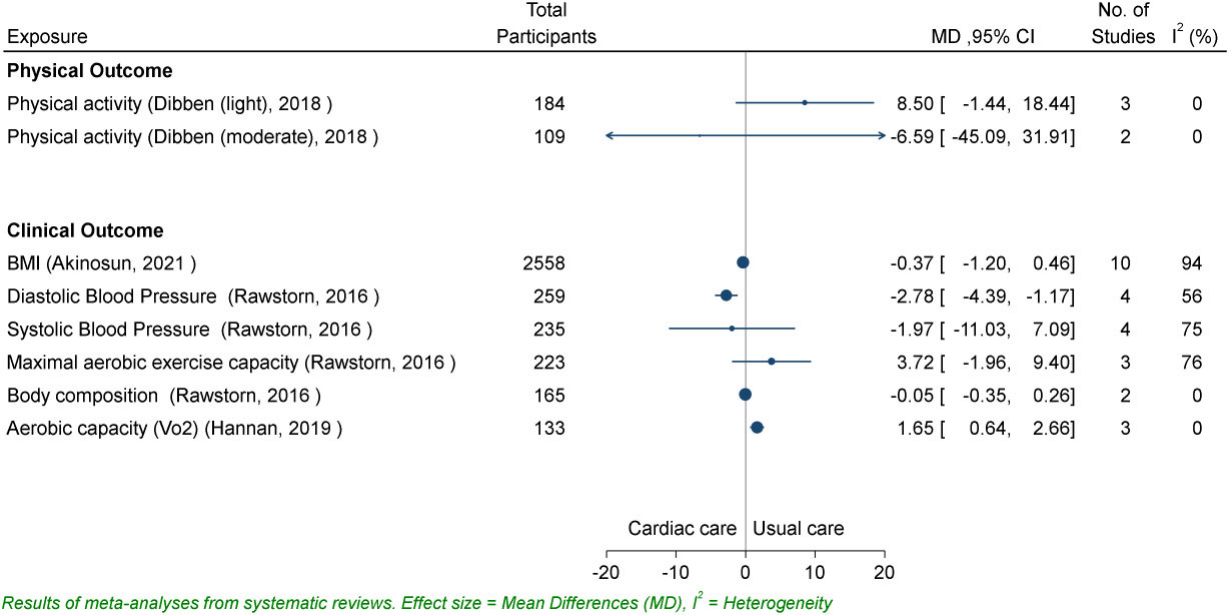
Mean difference for physical activity and clinical outcomes.

**Figure 3:**
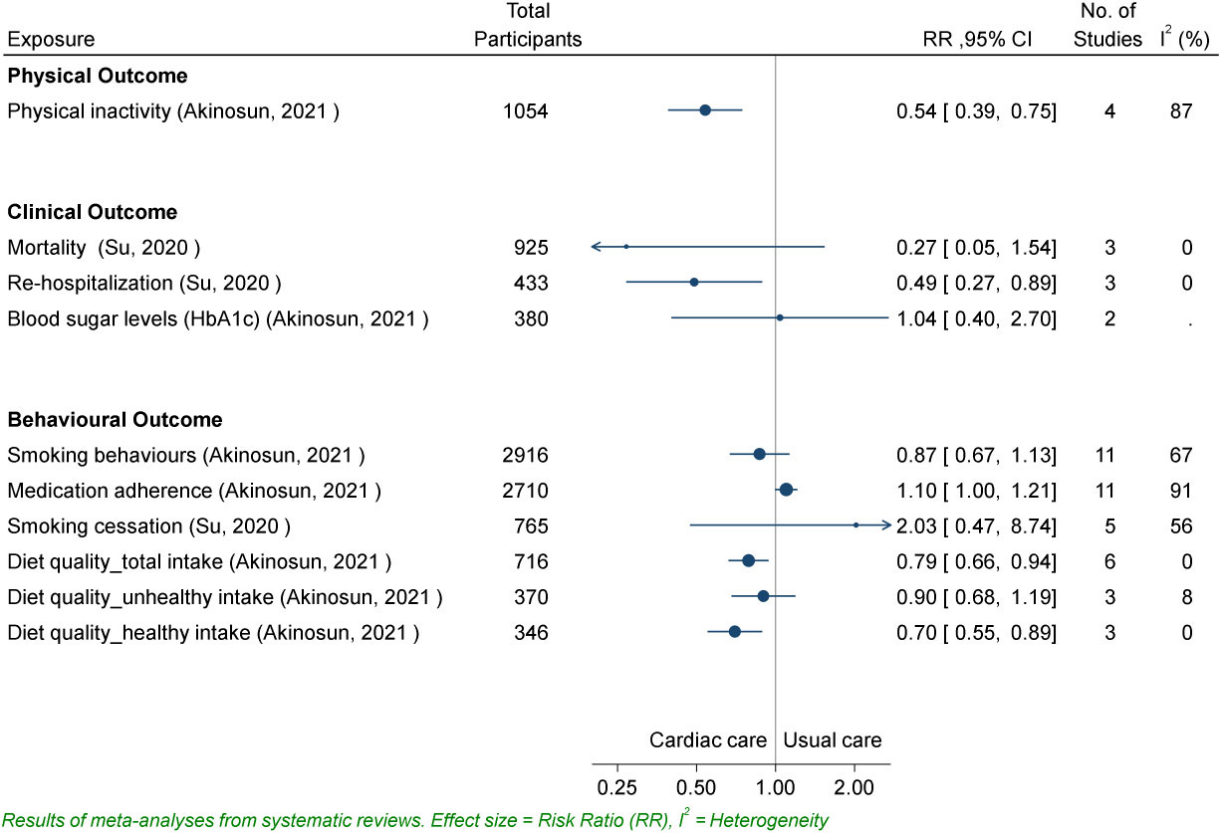
Relative risk for physical activity, clinical, and behavioural outcomes.

In *Figure* *2*, Dibben *et al.* (2018) reports the pooled mean minutes of both light and moderate level intensity, finding small effects. Moderate activity decreased [mean difference (MD) −6.59, 95% CI −45.09 to 31.91; *P* = 0.34] and light activity increased (MD 8.50, 95% CI −1.44 to 18.44; *P* = 0.68) for participants who engaged in CR interventions.^6^ In *Figure* *3*, the relative risk outcomes for physical inactivity described by Akinosun *et al.* (2021) indicated a significant reduction in sedentariness for CR patients (RR 0.54, 95% CI 0.39–0.75; *P* < 0.01)^23^ compared with usual care. For measures of duration of activity, step count and physical activity level, all four reviews found significant standardized mean increase in minutes of duration [standardized mean difference (SMD) 0.23, 95% CI 0.10–0.35; *P* < 0.01],^23^ step count (SMD 0.45, 95% CI −0.17 to 1.07; *P* = 0.15),^13^ and activity level (SMD 0.29, 95% CI 0.07–0.51; *P* < 0.01),^15^ indicating small to moderate effects and a high degree of agreement on the positive impact of CR interventions on physical activity.

Moderate to high heterogeneity (*I*^2^ > 50%) was present in four of the eight meta-analysis outcomes reported.^13,15,23^

#### The impact of devices/apps as part of CR on clinical outcomes

**Figure 4:**
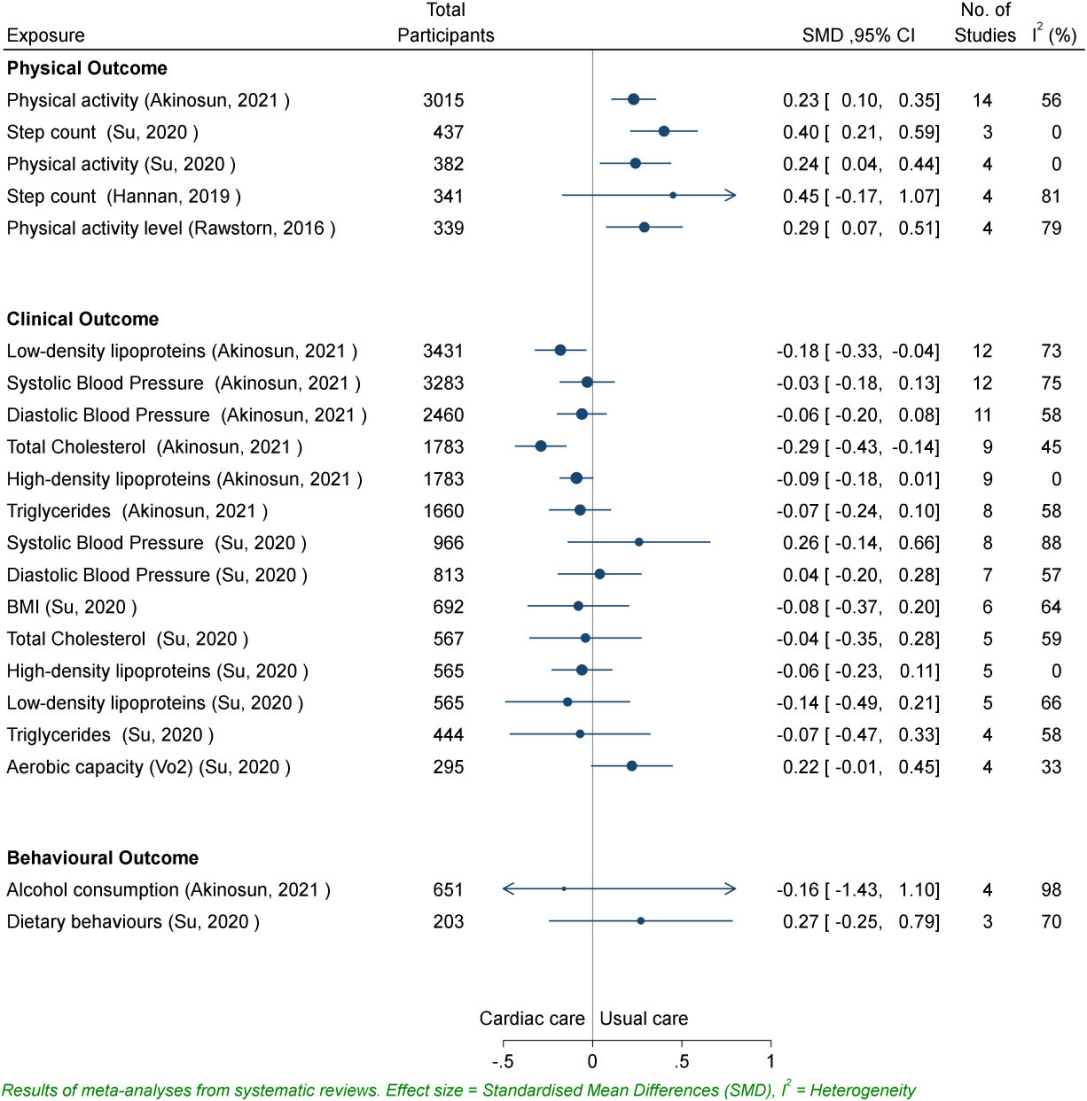
Standardized mean difference for physical activity, clinical, and behavioural outcomes.

A range of clinical outcomes were reported across all the nine reviews, with 23 clinical outcomes meta-analyses reported in four review articles^13,15,23,24^ (*Figures**2**–**4*). There was consistency in the direction of the outcomes, with mostly improved scores reported, including reductions in blood pressure, low and high-density lipoproteins, cholesterol, triglycerides, and BMI, and increases in aerobic capacity. However, all effect sizes for these outcomes were small, and authors acknowledged that such changes are unlikely to be clinically meaningful. For some measures, small sample size precludes conclusions about treatment effects.

Re-hospitalization rates and mortality risk^24^ indicated a risk reduction of 0.49 (*n* = 3, 95% CI 0.27–0.89; *P* = 0.02) and 0.27 (*n* = 5, 95% CI 0.05–1.54; *P* = 0.15), respectively, in CR group. Two reviews reported significant improvements for low-density lipoproteins (SMD −0.18, 95% CI −0.33 to −0.04; *P* < 0.01),^23^ high-density lipoproteins (SMD −0.09, 95% CI −0.18 to 0.01; *P* = 0.25),^23^ total cholesterol (SMD −0.29, 95% CI −0.43 to −0.14; *P* < 0.01),^23^ and triglycerides (SMD −0.07, 95% CI −0.24 to 0.10; *P* = 0.28).^23^ Systolic and diastolic blood pressure were significantly increased in one review^24^ and significantly reduced in two.^15,23^ The review with the greatest number of included studies reported an effect size for systolic blood pressure as −0.03 (SMD −0.03, 95% CI −0.18 to 0.13; *P* = 0.74) and −0.06 (SMD −0.06, 95% CI −0.20 to 0.08; *P* = 0.43) for diastolic^23^ intervention group compared with usual care group. Reductions in BMI were identified in two reviews, (SMD −0.08, 95% CI −0.37 to 0.20; *P* = 0.57)^24^ and (MD −0.37, 95% CI −1.20 to 0.46; *P* = 0.57),^24^ and in measures of body composition (body mass, waist, and hip circumferences) were reported in one (MD −0.05, 95% CI −0.35 to 0.26; *P* = 0.76).^15^ Blood glucose levels increased in the one review that reported this outcome (RR 1.04, 95% CI 0.40–2.70; *P* = 0.94).^23^ Intervention group participants’ aerobic capacity increased in the three meta-analysis that reported outcomes.^13,15,24^ For two reviews, the effect was positive but modest and non-significant.^15,24^ For Hannan *et al.* (2019), the small observed increase was significant in favour of wearables (MD 1.65, 95% CI 0.64–2.66; *P* < 0.01).^13^

Moderate heterogeneity (*I^2^* > 50%) was present in 14 of the 23 reported meta-analyses.^13,15,23,24^

#### The impact of devices/apps as part of CR on behavioural outcomes

Two reviews reported on behavioural outcomes. Akinosun *et al.* (2021) and Su *et al.* (2020) summarized outcomes on smoking behaviours and smoking cessation, diet quality, and dietary behaviours.^23,24^ Su *et al.* (2020) also described the impact of CR with apps on medication adherence.^24^ Inconsistencies in measures and modes of analysis limits the collation of effect sizes for these outcomes.

There was a common trend of improvements in health behaviours amongst intervention participants across the self-reported measures; however, many of these outcomes failed to reached significance. The mean alcohol intake was reduced as a result of the CR interventions.^23^ Based on five RCTs, Su *et al.* (2020) found the likelihood of smoking cessation increased (RR 2.03, 95% CI 0.47–8.74; *P* = 0.34),^24^ and Akinosun *et al.* (2021) found the percentage of people who identified as current smokers decreased (RR 0.87, 95% CI 0.67–1.13; *P* = 0.30).^23^ Medication adherence, measured using the Morisky Medication Adherence Scale, improved (RR 1.10, 95% CI 1.00–1.21; *P* = 0.06).^23^

Self-reported measures of diet quality and dietary behaviours indicate some significant improvement for participants in the intervention groups.^23,24^ Su *et al.* (2020) reported a very small and non-significant improvements in mean diet scores (SMD 0.27, 95% CI −0.25 to 0.79; *P* = 0.31).^24^ In the review by Akinosun *et al.* (2021), three measures of food intake were reported separately to indicate the individual treatment effects of each approach more clearly. Strategies to decrease overall intake (RR 0.79, 95% CI 0.66–0.94; *P* < 0.01) and increase healthy food intake (RR 0.70, 95% CI 0.55–0.89; *P* < 0.01) were successful and significant for intervention cohorts.^23^ Strategies to reduce unhealthy food intake resulted in a non-significant improvement (RR 0.90, 95% CI 0.68–1.19; *P* = 0.47).^23^

Quality of life meta-analysis outcomes, collected using a range of validated questionnaires, were reported in one review^24^ but were considered beyond the scope of this umbrella review. CR interventions were shown to have a strong, significant improvement in participants quality of life (SMD 0.95, 95% CI 0.12–1.79; *P* = 0.02).^24^ For the behavioural outcomes, moderate heterogeneity (*I^2^* > 50%) was present in five out of eight meta-analyses.^23,24^

## Discussion

This umbrella review suggests devices and applications offer some benefits to CR participants in terms of physical activity, clinical outcomes, and health behaviours. The trend in outcomes across all meta-analyses across all reviews was consistent, showing increases in activity and aerobic health, decreases in clinical measures and outcomes, reductions in unhealthy behaviours, and improvements in healthy behaviours, with similar effect sizes irrespective of the devices or applications used. However, most reported effects would have negligible clinical importance, and many were non-significant. Despite the lack of significance for many findings, the evidence-based narrative presents devices as effective at encouraging CR patients to increase their activity when used in conjunction with any mode of CR programme. Devices also appear to enhance exercise intensity during the maintenance phase of CR when used with exercise prescription and advice.^13^

A noted similarity across each of the reviews was the relatively short duration of follow-up, with study periods most commonly 1 to 6 months where study duration until follow-up was reported. This limits the evidence of effectiveness to short-term outcomes.^28^ Activity-monitoring devices and applications utilized in reviews varied widely, depending on the objectives of the studies, methodologies for collecting data, and the interventions used to educate and motivate participants. None of the reviews compared different types of monitoring devices and applications and did not collect evidence that would allow for comparisons across devices.

A very recent umbrella review investigating the effectiveness of activity monitoring devices for improving physical activity for all populations found similar uniformity in improvements in activity and clinical outcomes as this review, with modest, and often insignificant, effect sizes.^29^ Additionally, the authors also noted the broad consistency in outcomes despite the wide variety in metrics used to measure activity and health outcomes as an indication of the robustness of the results. Ferguson *et al.* (2022) recommend the use of devices, indicating the impact of devices, when used as part of programmes to increase physical activity, was positive, clinically important, and sustained for at least 6 months.

### Strengths and limitations

The systematic search strategy identified moderate- to high-quality reviews that reported a diverse range of physical activity measures and behavioural and clinical outcomes. However, review authors noted that RCT often lacked rigorous study design,^24^ and inconsistency in activity measures made it difficult to compare results.^22,23^ There was a modest overlap of primary studies across the included reviews, which is unlikely to bias the findings. The methodologies for measuring physical activity and behavioural outcomes varied, limiting the ability to make comparisons on outcomes reported across reviews. Devices and applications used to monitor physical activity also varied and included RCTs collecting self-reported activity measures, reducing the capacity to conduct meta-analyses. The accuracy and reliability of the data collected by devices and applications are unknown.

All included reviews reported the impact of devices and applications utilized during CR on physical activity and other outcomes. Of these, only two reviews directly compared the outcomes of intervention groups who completed CR programmes that utilized devices and applications to control groups who completed CR without devices or applications.^15,22^ The remainder compared CR programmes with devices and applications to controls who did not participate in a formal CR programme. To maximize the reporting for this umbrella review, all reviews that reported meta-analyses were described in the results, regardless of the control group. The possible effect of this inclusion could be an overestimation of effect sizes, as CR may explain some of the variance presented.

### Implications for practice and research

Our results suggest that using activity monitoring devices within CR programmes may improve physical activity levels and affect positively changes in other behaviours, particularly in telerehabilitation and programmes that include remote monitoring. Determining the effectiveness and potential for devices and applications to support CR is of growing importance as telerehabilitation programmes and home-based CR programmes become increasingly offered as an accessible and cost-effective alternative to traditional centre-based CR.^22^ Given the lack of evidence on the superiority of a specific device found by this review and as guidelines recommendations emphasize the duration and intensity of the physical activity,^2,3,5^ it is reasonable that devices that measure these components be considered by CR programmes. For successful translation of these findings into practice, patient preferences and the local context of the CR programme should be considered. Key aspects for implementation would include ensuring equity in access to the devices, burden on clinicians, seamless integration of the data collected by the devices within existing systems in a secure manner, and training on how to optimize the use of the data to provide feedback to patients. These, along with the need of long-term outcomes and economic evaluations, should be considered by implementation research to allow patients to benefit from these technologies in real-world settings.

## Conclusions

The outcomes reported in the included systematic reviews indicate overall improvement in physical activity, clinical, and healthy eating behaviours when using activity-monitoring devices and mobile applications integrated into CR. The summary of findings endorsed the utilization of devices as a practical addition to all modes of CR, but particularly telerehabilitation and programmes that include remote monitoring.

## Supplementary Material

qcad005_Supplemental_File

## Data availability

All data are incorporated into the article and its online supplementary material.
