# Supplementary material for: Cardiac rehabilitation, physical activity, and the effectiveness of activity monitoring devices on cardiovascular patients: an umbrella review of systematic reviews
Source: Eur Heart J Qual Care Clin Outcomes. 2023 Jan 23;9(4):323–30. doi: 10.1093/ehjqcco/qcad005 (PMC10284262; doi:10.1093/ehjqcco/qcad005)
Supplement: qcad005_Supplemental_File [file qcad005_supplemental_file.docx]

**Appendices**

**Appendix 1:**

**Supplementary Table 2: Characteristics of included studies**

| Authors | Number of studies (N) | Number of participants (N) | Sex Male % | Mean Age (years) | Diagnosis | Study duration (weeks) | Follow-up  (weeks) | Number of Events  Intervention / Control | Objectives | Type of wearables | Intervention description | | Outcome and results |
| --- | --- | --- | --- | --- | --- | --- | --- | --- | --- | --- | --- | --- | --- |
|  |  |  |  |  |  |  |  |  |  |  | Control | Intervention |  |
| Akinosun, 2021 | 25 | 5,779 | 75.2 | 60 | CVD, CAD, MI, ACSA  atherosclerosis, HF, TIA,  stroke. | 4 | NS | NS/NS | To assess the effectiveness of digital technology to modify risk factors and behaviour change in patients with CVD | NS | Usual care | CR with internet and / or mobile applications and mobile sensors | Behavioural (PA, PI, diet, smoking, alcohol, MA)  Clinical (BMI, CL, TC, HDLs, LDLs, TGs, BP, HbA1c) |
| Hannan, 2019 | 9 | 1,352 | 77.9 | 42-74 | MI, CA bypass graft surgery, PCI, ACS, CAD, CABG, VR, HF, UA | 6-72 | NS | NS/NS | To measure the psychological effect of WPAM with exercise prescription during the maintenance phase of CR and adverse events,  and psychological impact of CR utilising a WPAM | Yamax Digiwalker  Pedometer, Garmin Forerunner, Fitbit Charge, My Wellness Key Accelerometer,  Gex vital signs sensor, Nokia Smartphone  application, Sensewear Mini Armband | Usual care post CR | CR monitoring with wearable PA tracking devices | Behavioural (step count, exercise intensity, PI)  Clinical (CRF, EC, EDQ5, DASS 21, Kessler 6, QoL) |
| Batalik, 2020 | 12 | 545 | NS | 60 | CVD, HF, MI | 6-24 | Up to 52 | NS/NS | To assess the efficiency, utilisation and safety of telerehabilitation with remotely monitoring | Chest strap HR monitor  Biometric vest, belt strap sensor, accelerometer, wearable ECG mobile phone app, chest worn wearable ECG sensor, HR monitor, ECG, BP, body weight monitor, ECG sensor vest, step counter, BP and body weight monitor | Centre-based CR | CR telerehabilitation with remote monitoring using wearable BP, HR tracking devices | Behavioural (diet, smoking, exercise motivation)  Clinical (EC, EE, psychosocial functioning, BP, HR, weight, BMI, WC, LDL, HbA1c) |
| Indraratna, 2020 | 8 | 735 | NS | NS | IHD, ACS, HF, HTN, CM | 6-52 | NS | NS | To review the evidence for mobile phone technology in the management of cardiac conditions, in relation to the data clinicians can access and patient clinical outcomes and risk factors | Mobile phone  Sphygmomanometer, weighing scale, ECG monitor/ECG monitoring vest, step-counter | Usual care | CR with mobile phone support and remote monitoring using HR, wearable PA tracking devices | Behavioural (CR Completion, MA, Lifestyle)  Clinical (PF, FS,  mortality, hospitalisation) |
| Marin, 2019 | 10 | 849 | 72.3 | 42-82 | ACSA, HF, CAD, MI | NS | 4-52 | NS | The measure the extent of adherence in the use of activity-monitoring devices, the impact of activity-monitoring devices on activity levels; and the perceived acceptability of activity-monitoring devices | Accelerometer, pedometer | Usual care | CR within a community setting with monitoring using activity tracking devices | Behavioural (Adherence to program, adherence to device, PA, steps, EE, user acceptability) |
| Su,  2020 | 14 | 1783 | 78.5 | 60 | CAD, HF, MI, other CVD. | 6-60 | 13-52 | Rehospitalization: 14/28  Mortality: 0/4. | To evaluate the effects of eHealth CR on health outcomes of CHD patients and to identify programme design, which may lead to more effective health benefits | NS | Usual care | CR with website and / or mobile applications support | Behavioural (PA, diet, smoking cessation, MA)  Clinical (BP, TC, HDL, LDL, TGs, Weight, QoL, V̇O2 max, Re-hospitalisation, mortality) |
| Rawstorn, 2016 | 11 | 1189 | 75 | 58 | CAD(atherosclerosis, AP, MI, coronary revascularisation) | 6.5-52 | Up to 26 | NS | To determine the effectiveness and safety of structured telehealth exercise-based CR on maximal aerobic exercise capacity and modifiable cardiovascular risk factors compared with traditional centre-based exercise-based CR and usual care | NS | Usual care, Centre-based exercise CR | Telerehabilitation with information and communications technology (telephone, smartphone, mobile applications, computer, Internet, and / or biosensors) for structured exercise training | Behavioural (PA level, EA)  Clinical (BP, Blood lipids, body composition, blood glucose, clinical events) |
| van Veen, 2017 | 19 | NS | 70 | 67 | HF, CVD, CAD, IHD, MI, CABG | 6 to 52 weeks | 0.3-76.5 | NS | To provide an evidence-based overview of the effectiveness of e-coaching as a CR program | NS | Usual care | Web-based or telerehabilitation self-management CR programs with website and / or mobile applications for support and home-based monitoring | Behavioural (Adherence, PA)  Clinical (BP, cholesterol; hospital visits, psychosocial outcomes, patient feedback) |
| Dibben, 2018 | 40 | 6480 | NS | 58 | HF, CAD. | NS | 6.5-516 | NS | To undertake a systematic review and meta-analysis to assess the impact of CR on physical activity levels of patients with heart disease and the methodological quality of these studies. | Accelerometer, Pedometer | Usual care | CR with an exercise component and monitoring of PA | Behavioural (Steps, EE, PI, PA) |

Study design – For all SRs it was RCT

Abbreviations: ACSA: acute coronary syndrome angina; AP: angina pectoris; ACS: acute coronary syndrome; BP: blood pressure; BMI: body mass index; CA: coronary artery; CABG: coronary artery bypass graft; CAD: coronary artery disease; CF: cardiac failure; CL: cholesterol levels; CM: cardiomyopathy; CR: cardiac rehabilitation; CRF: cardiorespiratory fitness; CVD: cardiovascular diseases; DASS 21: decreased depression, anxiety and stress scale; EA: exercise adherence; EC: exercise capacity; EDQ5: general health status scale; EE: energy expenditure; FS: functional status; HDL: high density lipoprotein; HF: heart failure; HR: heart rate; HTN: hypertension; LDL: low density lipoprotein MA: medication adherence; MI: myocardial infarction; NS: not specified; PA: physical activity; PCI: percutaneous coronary intervention; PF: physical fitness; PI: physical inactivity; QoL: quality of life; TC: total cholesterol; TG: thyroglobulin; TIA: transient ischemic attack; UA: unstable angina; VR: valve replacement; WC: weight control

**Appendix 2:**

**Supplementary Table 3: Excluded studies and reason for exclusion**

|  | **Excluded studies** | **Reason for exclusion** |
| --- | --- | --- |
| 1. | Akinosun AS, Polson R, Diaz - Skeete Y, De Kock JH, Carragher L, Leslie S, et al. Digital Technology Interventions for Risk Factor Modification in Patients With Cardiovascular Disease: Systematic Review and Meta-analysis. JMIR Mhealth Uhealth [Internet]. 2021;9(3):e21061. | Duplicated paper |
| 2. | Allida S, Du H, Xu X, Prichard R, Chang S, Hickman LD, et al. mHealth education interventions in heart failure. Cochrane Database of Systematic Reviews [Internet]. 2020;(7). | Doesn’t meet criteria |
| 3. | Amanda H. Impact of wearable physical activity monitoring devices with exercise prescription or advice in the maintenance phase of cardiac rehabilitation: systematic review and meta-analysis. BMC sports science, medicine & rehabilitation. 2019;11(1):N.PAG. | Duplicated paper |
| 4. | Anonymous. Combined Sections Meeting of the American Physical Therapy Association. Cardiopulmonary Physical Therapy Journal Conference: Combined Sections Meeting of the American Physical Therapy Association, CSM [Internet]. 2021;32(3). | Doesn’t meet criteria |
| 5. | Ashur C, Cascino T, Lewis C, Richardson C, Jackson E. Wearable Technology as an Intervention for Patients with Coronary Heart Disease in an Exercise-Based Rehabilitation Program: A Systematic Review. Journal of the American College of Cardiology (JACC) [Internet]. 2019;73(9):1827–1827. | Record not retrieved |
| 6. | Bamforth RJ, Chhibba R, Ferguson TW, Sabourin J, Pieroni D, Askin N, et al. Strategies to prevent hospital readmission and death in patients with chronic heart failure, chronic obstructive pulmonary disease, and chronic kidney disease: A systematic review and meta-analysis. PLoS ONE [Internet]. 2021;16 (4 April) (no pagination). | Doesn’t meet criteria |
| 7. | Bhavnani S, Waalen J, Srivastava A, Heywood JT. Which patients? which devices? mhealth monitoring with wearable and implantable devices in heart failure: Meta analyses of randomized trials. Journal of the American College of Cardiology [Internet]. 2015;1): A1030. | Record not retrieved |
| 8. | Chase J-AD. Systematic review of physical activity intervention studies after cardiac rehabilitation. Journal of Cardiovascular Nursing [Internet]. 2011;26(5):351–8. | The paper doesn't focus on physical activity monitoring in CR patients |
| 9. | Gandhi S, Hong L, Sun K, Schwalm J. Effect of mobile health interventions on the secondary prevention of cardiovascular disease: Systematic review and meta-analysis. Canadian Journal of Cardiology [Internet]. 2016;32(10 9. Supplement 1):S278. | Doesn’t meet criteria |
| 10. | Halldorsdottir H, Thoroddsen A, Ingadottir B. Impact of technology-based patient education on modifiable cardiovascular risk factors of people with coronary heart disease: A systematic review. Patient Education and Counseling [Internet]. 2020;103(10):2018–28. | The paper doesn't focus on physical activity monitoring in CR patients |
| 11. | Hannan AL, Harders MP, Hing WY, Climstein M, Coombes JS, Furness J. Impact of wearable physical activity monitoring devices with exercise prescription or advice in the maintenance phase of cardiac rehabilitation: systematic review and meta-analysis. BMC Sports Science Medicine and Rehabilitation [Internet]. 2019;11. | Duplicated paper |
| 12. | Hodkinson A, Kontopantelis E, Adeniji C, Van Marwijk H, McMillian B, Bower P, et al. Interventions using wearable physical activity trackers among adults with cardiometabolic conditions A systematic review and meta-analysis. JAMA Network Open. 2021;4(7). | Doesn’t meet criteria |
| 13. | Hodkinon A, Kontopantelis E, Adeniji C, van Marwijk H, McMillan B, Bower P, et al. Accelerometer- and Pedometer-Based Physical Activity Interventions Among Adults With Cardiometabolic Conditions A Systematic Review and Meta-analysis. JAMA Network Open [Internet]. 2019;2(10). | The paper doesn't focus on outpatient cardiac rehabilitation |
| 14. | Indraratna P, Tardo D, Yu J, Brodie M, Delbaere K, Lovell N, et al. 769 Mobile Phone Interventions in Heart Failure - a Systematic Review and Meta-Analysis. Heart Lung and Circulation [Internet]. 2020;29(Supplement 2): S382–3. | Doesn’t meet criteria |
| 15. | Inglis SC, Clark RA, McAlister FA, Stewart S, Cleland JG. Which components of heart failure programmes are effective? A systematic review and meta-analysis of the outcomes of structured telephone support or telemonitoring as the primary component of chronic heart failure management in 8323 patients: Abridged Cochrane Review. European Journal of Heart Failure [Internet]. 2011;13(9):1028–40. | The paper doesn't focus on outpatient cardiac rehabilitation |
| 16. | Jiang X, Ming WK, You JH. The Cost-Effectiveness of Digital Health Interventions on the Management of Cardiovascular Diseases: Systematic Review. Journal of Medical Internet Research. 2019;21(6):e13166. | The paper doesn't focus on outpatient cardiac rehabilitation |
| 17. | Jolliffe J, Taylor R. Physical activity and cardiac rehabilitation: a critical review of the literature. Coronary Health Care [Internet]. 1998;2(4):179–86. | Doesn’t meet criteria |
| 18. | Marin TS, Kourbelis C, Foote J, Newman P, Brown A, Daniel M, et al. Examining adherence to activity monitoring devices to improve physical activity in adults with cardiovascular disease: A systematic review. European Journal of Preventive Cardiology [Internet]. 2019;26(4):382–97. | Duplicated paper |
| 19. | Martha Kyriakou M, Klompstra L, Jaarsma T, Middleton N, Piepoli M, Lambrinou E. Devices for objective assessment of physical activity in patients with heart failure: A systematic review. European Journal of Heart Failure [Internet]. 2018;20(Supplement 1):583. | Doesn’t meet criteria |
| 20. | Muzas Fernández A, Soto González M. Utilización de smartphone en los programas de rehabilitación cardíaca. Una revisión sistemática. Rehabilitacion [Internet]. 2018;52(4):238–45. | Duplicated paper |
| 21. | Piotrowicz E, Piotrowicz R. Telemonitoring in heart failure rehabilitation. European Cardiology [Internet]. 2011;7(1):66–9. | The paper is not a SR paper |
| 22. | Su JJ, Yu DSF, Paguio JT. Effect of eHealth cardiac rehabilitation on health outcomes of coronary heart disease patients: A systematic review and meta-analysis. Journal of Advanced Nursing [Internet]. 2020;76(3):754–72. | Duplicated paper |
| 23. | Turan Kavradim S, Özer Z, Boz İ. Effectiveness of telehealth interventions as a part of secondary prevention in coronary artery disease: a systematic review and meta‐analysis. Scandinavian Journal of Caring Sciences [Internet]. 2020;34(3):585–603. | The paper doesn't focus on outpatient cardiac rehabilitation |
| 24. | Verburg A, Selder JL, Schalij MJ, Schuuring MJ, Treskes RW. eHealth to improve patient outcome in rehabilitating myocardial infarction patients. Expert Review of Cardiovascular Therapy [Internet]. 2019;17(3):185–92. | The paper is not a systematic review |
| 25. | Wongvibulsin S, Habeos EE, Huynh PP, Xun H, Shan R, Rodriguez KAP, et al. Digital Health Interventions for Cardiac Rehabilitation: Systematic Literature Review. Journal of Medical Internet Research [Internet]. 2021;23(2). | Doesn’t meet criteria |
| 26. | Wongvibulsin S, Habeos EE, Huynh PP, Xun H, Shan RZ, Rodriguez KAP, et al. Digital Health Interventions for Cardiac Rehabilitation: Systematic Literature Review. Journal of Medical Internet Research [Internet]. 2021;23(2). | Doesn’t meet criteria |
| 27. | Munro J, Angus N, Leslie SJ. Patient focused internet-based approaches to cardiovascular rehabilitation–a systematic review. Journal of Telemedicine and Telecare. 2013;19(6):347–53. | Doesn’t meet criteria |
